# Impact of the type of mask on the effectiveness of and adherence to continuous positive airway pressure treatment for obstructive sleep apnea*

**DOI:** 10.1590/S1806-37132014000600010

**Published:** 2014

**Authors:** Rafaela Garcia Santos de Andrade, Vivien Schmeling Piccin, Juliana Araújo Nascimento, Fernanda Madeiro Leite Viana, Pedro Rodrigues Genta, Geraldo Lorenzi-Filho

**Affiliations:** University of São Paulo, School of Medicine, Hospital das Clínicas, São Paulo, Brazil. Sleep Laboratory, Department of Pulmonology, Heart Institute, University of São Paulo School of Medicine Hospital das Clínicas, São Paulo, Brazil; University of São Paulo, School of Medicine, Heart Institute, São Paulo, Brazil. Sleep Laboratory, Department of Pulmonology, Heart Institute, University of São Paulo School of Medicine Hospital das Clínicas, São Paulo, Brazil; University of São Paulo, School of Medicine, Hospital das Clínicas, São Paulo, Brazil. Sleep Laboratory, Department of Pulmonology, Heart Institute, University of São Paulo School of Medicine Hospital das Clínicas, São Paulo, Brazil; University of São Paulo, School of Medicine, Hospital das Clínicas, São Paulo, Brazil. Sleep Laboratory, Department of Pulmonology, Heart Institute, University of São Paulo School of Medicine Hospital das Clínicas, São Paulo, Brazil; University of São Paulo, School of Medicine, Hospital das Clínicas, Boston, MA, USA. Sleep Laboratory, Department of Pulmonology, Heart Institute, University of São Paulo School of Medicine Hospital das Clínicas, São Paulo, Brazil; and Postdoctoral Student, Division of Sleep Medicine, Brigham and Women's Hospital/Harvard University, Boston (MA) USA; University of São Paulo, School of Medicine, Hospital das Clínicas, São Paulo, Brazil. Sleep Laboratory, Department of Pulmonology, Heart Institute, University of São Paulo School of Medicine Hospital das Clínicas, São Paulo, Brazil

**Keywords:** Sleep apnea, obstructive, Continuous positive airway pressure, Masks

## Abstract

Continuous positive airway pressure (CPAP) is the gold standard for the treatment of obstructive sleep apnea (OSA). Although CPAP was originally applied with a nasal mask, various interfaces are currently available. This study reviews theoretical concepts and questions the premise that all types of interfaces produce similar results. We revised the evidence in the literature about the impact that the type of CPAP interface has on the effectiveness of and adherence to OSA treatment. We searched the PubMed database using the search terms "CPAP", "mask", and "obstructive sleep apnea". Although we identified 91 studies, only 12 described the impact of the type of CPAP interface on treatment effectiveness (n = 6) or adherence (n = 6). Despite conflicting results, we found no consistent evidence that nasal pillows and oral masks alter OSA treatment effectiveness or adherence. In contrast, most studies showed that oronasal masks are less effective and are more often associated with lower adherence and higher CPAP abandonment than are nasal masks. We concluded that oronasal masks can compromise CPAP OSA treatment adherence and effectiveness. Further studies are needed in order to understand the exact mechanisms involved in this effect.

## Abstract (pt)

O tratamento com *continuous positive airway pressure* (CPAP, pressão positiva continua nas vias aéreas) é o padrão ouro na apneia obstrutiva do sono (AOS). Apesar de a CPAP ter sido originalmente aplicada através de máscara nasal, várias interfaces são atualmente disponíveis. Revisamos conceitos teóricos e questionamos a premissa de que todas as máscaras produzem resultados semelhantes. Compilamos as evidências na literatura sobre o impacto do tipo de máscara na eficácia e na adesão ao tratamento com CPAP em pacientes com AOS. Foram pesquisados artigos escritos em inglês na base de dados PubMed com as palavras-chave "*CPAP*", "*mask*" e "*obstructive sleep apnea*". Dos 91 artigos encontrados, somente 12 foram selecionados por descrever o impacto do tipo de máscara sobre a efetividade (n = 6) ou a adesão (n = 6) ao tratamento. Apesar de alguns resultados conflitantes, não encontramos efeitos significativos da máscara *pillow* nasal e da máscara oral na eficácia ou adesão ao tratamento da AOS. Em contraste, a maior parte dos estudos mostrou que a máscara oronasal é menos efetiva e mais frequentemente associada a menor adesão e maior abandono do tratamento do que a máscara nasal. Concluímos que a máscara oronasal pode comprometer a eficácia e a adesão ao tratamento da AOS com CPAP. Estudos futuros são necessários para a compreensão dos mecanismos exatos envolvidos neste efeito.

## Introduction

Obstructive sleep apnea (OSA) is characterized by repeated episodes of partial pharyngeal obstruction (hypopnea) or complete pharyngeal obstruction (apnea) associated with oxygen desaturation and sleep fragmentation.^(^ 1 ^,^ 2 ^)^ Polysomnography is the gold standard for the diagnosis of OSA, and the main parameter is the apnea-hypopnea index (AHI), which indicates the number of apnea and hypopnea events per hour of sleep. In a recent study, in which a representative sample of patients in the city of São Paulo, Brazil, underwent polysomnography (n = 1,042), it was found that approximately one in every three adults (32.8%) met the criteria for OSA syndrome, characterized by an AHI of more than 5 events/hour of sleep with symptoms or an AHI of more than 15 events/hour of sleep with or without symptoms.^(^ 3 ^)^ The consequences of OSA are many, including sleep fragmentation, nonrestorative sleep, excessive daytime sleepiness, impaired quality of life, and increased cardiovascular complications, such as systemic arterial hypertension, cardiac arrhythmia, and increased risk of mortality.^(^ 4 ^-^ 9 ^)^

Application of continuous positive airway pressure (CPAP) during sleep is the gold standard for the treatment of patients with moderate to severe OSA. In patients with OSA, treatment with CPAP can reduce excessive daytime sleepiness,^(^ 10 ^,^ 11 ^)^ improve cognitive function, improve quality of life,^(^ 11 ^)^ reduce blood pressure in those with hypertension, and reduce the risk of cardiovascular morbidity and mortality.^(^ 12 ^,^ 13 ^)^ The efficacy of the treatment depends on the use of CPAP each night during sleep.^(^ 14 ^)^ However, adherence to CPAP therapy is extremely variable (46-80%). ^(^ 15 ^,^ 16 ^)^ Predictors of adherence to CPAP therapy include the severity of OSA, the degree of daytime sleepiness, the socioeconomic status, the level of patient understanding of the therapy, and the type of mask used.^(^ 17 ^-^ 20 ^)^

**Figure 1 -:**
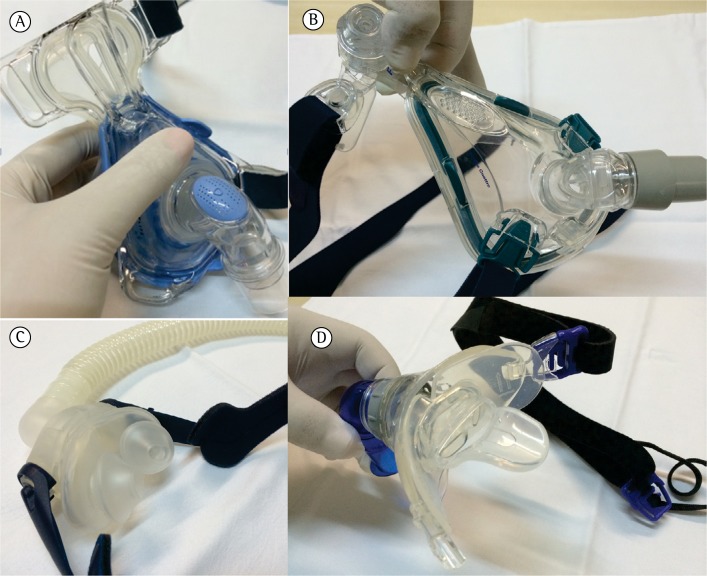
Photographs showing the types of continuous positive airway pressure masks currently available for the treatment of obstructive sleep apnea: nasal mask, in A; oronasal mask, in B; nasal pillows, in C; and oral mask, in D. Source: Sleep Laboratory, Heart Institute, University of São Paulo School of Medicine Hospital das Clínicas.

Treatment of OSA with CPAP was first described by Sullivan et al. in 1981.^(^ 21 ^)^ The key idea was that CPAP applied with a nasal mask acted as a pneumatic splint to maintain upper airway patency, moving the soft palate anteriorly. An increasing number of masks that are lighter and more comfortable are becoming available for use in patients with nasal obstruction. Currently available types of masks include nasal masks, nasal pillows, oronasal masks, and oral masks (Figure 1). Nasal masks cover only the nose and must surround it so as not to compress the nasal alae, sitting just above the upper lip and near the angle of the eye. Nasal pillows consist of two nasal inserts and have emerged as an alternative to nasal masks because they are smaller and have less contact with the face. Oronasal masks cover the nose and the mouth and allow patients to breathe through their nose and their mouth. Oronasal masks were initially described for noninvasive ventilation in patients with respiratory failure and high ventilatory demand. ^(^ 22 ^)^ Oronasal masks are considered an option for OSA patients with complaints of nasal obstruction and mouth breathing.^(^ 23 ^-^ 25 ^)^ Oral masks are made of silicone and resemble a butterfly, sitting between the lips and teeth. Oral masks include a tongue guide designed to hold the tongue in place and prevent it from blocking the flow of air from the CPAP. In clinical practice, oral masks are not widely used. With the objective of improving CPAP treatment adherence, a variety of materials are used in the manufacture of CPAP masks, including silicone, gel, and fabrics. A Google^(r)^ search returns approximately 1,600,000 results for the search terms "nasal mask", "oronasal mask", and "nasal pillows"; this illustrates the diversity of interfaces and materials that are currently available. Despite this diversity, the level of scientific evidence for the efficacy of new models and their impact on treatment adherence have been questioned. In the present review, we sought to answer two questions: Can the type of mask affect the efficacy of CPAP treatment for OSA? Can it influence adherence to CPAP treatment? 

## The impact of the type of interface on the efficacy of OSA treatment with CPAP

**Figure 2 -:**
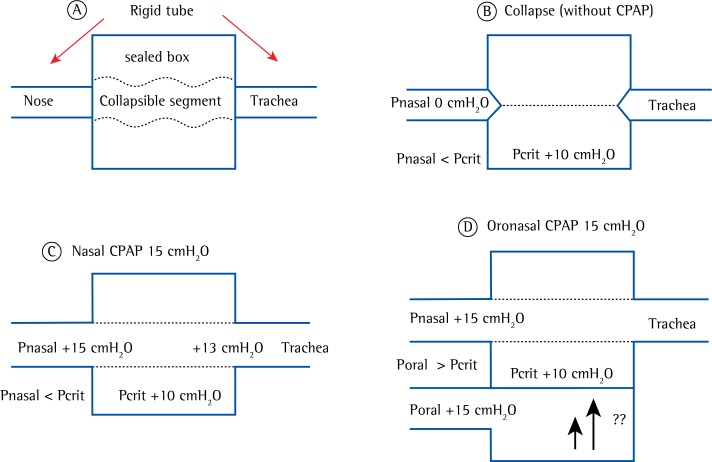
Schematic illustration of the Starling resistor. In A, the nose and the trachea are represented by two rigid tubes connected by a collapsible segment (the pharynx). In B, pharyngeal collapse occurs when the pharyngeal critical pressure (Pcrit) is greater than the upper airway pressure (Pnasal). In C, nasal continuous positive airway pressure (CPAP) applied to the upper airway is greater than Pcrit and can therefore maintain upper airway patency. In D, oronasal CPAP; the hypothesis is that upper airway collapse occurs when oral pressure (Poral) is greater than Pcrit. Source: Sleep Laboratory, Heart Institute, University of São Paulo School of Medicine Hospital das Clínicas.

**Figure 3 -:**
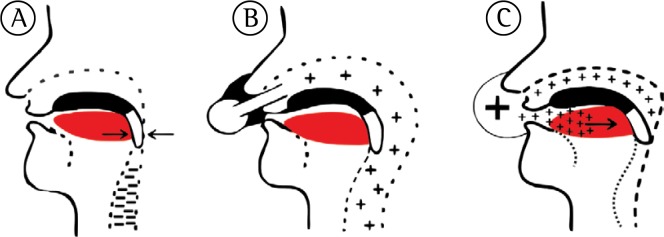
In A, schematic illustration of the normal upper airway (left) of a patient with obstructive sleep apnea, showing retropalatal obstruction during negative pressure generated during inhalation (center) and during continuous positive airway pressure (right). In B, schematic illustration of a patient wearing an oronasal mask, and, in C, patient with significant mouth breathing. The tongue (red) is displaced posteriorly and obstructs the upper airway. Adapted from Sullivan et al.(21) Source: Schorr et al.(31)

The mechanisms of airway obstruction in patients with OSA and the effects of CPAP can be explained by the Starling resistor model. The Starling resistor consists of two rigid tubes connected by a collapsible tube. The two rigid tubes represent the nose and the trachea, which are bony and cartilaginous structures. The pharynx, which is a collapsible, muscular tube, lies between the two. In this model, the pharyngeal critical pressure is the pressure at which complete pharyngeal collapse occurs.^(^ 26 ^)^ The trend toward pharyngeal collapse depends on nasal and tracheal pressure, as well as on the pressure surrounding the pharynx. The fundamental concept is that the pressure that nasal CPAP applies to the pharyngeal lumen is greater than the pharyngeal critical pressure, thus ensuring pharyngeal patency (Figure 2). It is of note that the Starling resistor model involves variations in nasal pressure. It is clear that the model assumes that the mask is nasal rather than oronasal. Accordingly, Sullivan et al. proposed that CPAP be applied via a nasal mask when they first described the treatment of OSA with CPAP.^(^ 21 ^)^ Although it works, the application of CPAP via an oronasal mask for the treatment of OSA violates the principles of the Starling resistor model (Figure 2) and those of the model originally described by Sullivan et al. (Figure 3). From a conceptual standpoint, the pressure that opens the pharynx when applied nasally can also lead to pharyngeal collapse when applied orally. 

**Figure 4 -:**
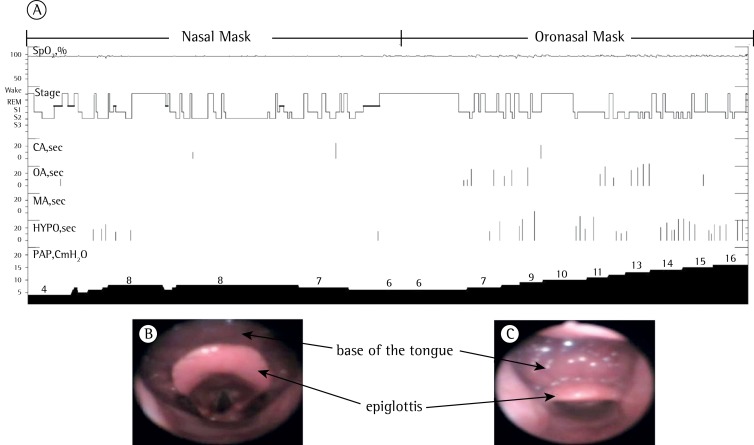
In A, polysomnography summary of a continuous positive airway pressure (CPAP) titration study during natural sleep. In B and C, sleep endoscopy images showing the patient wearing a nasal mask and an oronasal mask, respectively. During the first half of the CPAP titration study, a nasal CPAP of 7 cmH2O was enough to control obstructive events. During the second half of the CPAP titration study, an oronasal mask was used. Obstructive events persist, despite the fact that the pressure was gradually increased to 16 cmH2O. The endoscopic image obtained when a nasal CPAP of 7 cmH2O was used (B) shows that the oropharynx is open. In contrast, the image obtained when an oronasal CPAP of 16 cmH2O was used (C) shows posterior displacement of the base of the tongue, which pushes the epiglottis and significantly narrows the airway lumen. Respiratory events are expressed in seconds (sec). SpO2 measured by pulse oximetry. CA: central apnea; OA: obstructive apnea; MA: mixed apnea; Hypo: hypopnea; PAP: pulmonary artery pressure; and REM: rapid eye movement.

The efficacy of oronasal masks can be questioned on theoretical and experimental grounds. Upper airway resistance during sleep and the propensity for obstructive apneas are significantly greater during mouth breathing than during nasal breathing.^(^ 27 ^)^ A study of 6 patients with severe OSA^(^ 28 ^)^ showed that the pressure required to maintain upper airway patency with the use of a nasal mask was insufficient to maintain upper airway patency when an oronasal mask was used. A study of 11 patients with OSA showed that oropharyngeal resistance was higher with the use of an oronasal mask than with the use of a nasal mask or a nasal mask with a mandibular advancement device.^(^ 29 ^)^ The deleterious effect of the oronasal mask was reversed by concomitant use of the mandibular advancement device. Therefore, the authors of that study hypothesized that the increased resistance observed with the use of an oronasal mask was caused by posterior tongue displacement. This hypothesis was confirmed in a study of two patients with Down syndrome,^(^ 30 ^)^ in whom CPAP applied via an oronasal mask resulted in posterior tongue displacement and reduced upper airway patency. We have recently reported the case of a 69-year-old male patient with severe OSA and persistent sleepiness, despite adequate use of CPAP applied with an oronasal mask.^(^ 31 ^)^ We conducted an oronasal CPAP titration study and found a residual AHI of 32 events/hour of sleep, despite the fact that CPAP was gradually increased to 16 cmH_2_O. A new CPAP titration study confirmed that the interface was affecting the efficacy of CPAP; we found that a nasal CPAP of 7 cmH_2_O was enough to eliminate OSA in the first half of the study. The nasal mask was changed to an oronasal mask during the second half of the study, and the latter was found to be ineffective in eliminating OSA. In order to clarify the mechanisms involved in this apparent paradox, we conducted a CPAP titration study during midazolam-induced sleep.^(^ 31 ^)^ The mask was customized to allow passage of the endoscope for direct visualization of the oropharynx. As expected, a nasal CPAP of 7 cmH_2_O opened the oropharynx during sleep. In contrast, the oropharynx was found to be partially obstructed by posterior displacement of the base of the tongue with the use of an oronasal CPAP of 16 cmH_2_O (Figure 4).^(^ 32 ^)^ In that patient, oral CPAP caused posterior tongue displacement, which affected the efficacy of nasal CPAP. The case of that patient does not appear to be unique; our observations are couched in a solid theoretical framework and are corroborated by the findings of several experimental studies, prompting us to conduct the present literature review. 

## Methods

We searched the PubMed database using the search terms "CPAP", "mask", and "obstructive sleep apnea". The process of selection of articles for the review was divided into three stages. First, we selected the titles of interest. Second, we analyzed the abstracts of the selected articles to ensure that the studies addressed the impact of the type of CPAP mask on OSA treatment adherence, efficacy, or both. Although we thus identified 91 studies, only 12 met the inclusion criteria. A study comparing nasal and oronasal masks in only 5 patients receiving CPAP was inconclusive and was therefore not included in the final analysis.^(^ 33 ^)^ Of the 12 studies included in the review, 6 described the impact of the type of CPAP interface on treatment efficacy. Of those, 2 also addressed treatment adherence and were therefore analyzed in conjunction with the 6 studies describing the impact of the type of CPAP interface on treatment adherence. The included articles were divided into observational studies and randomized studies. 

## Impact of the type of mask on the efficacy of OSA treatment with CPAP

We found 6 studies evaluating the efficacy of CPAP masks in the treatment of OSA. Table 1 shows an overview of the studies. Of the 6 studies, 3 were observational studies^(^ 34 ^-^ 36 ^)^ and 3 were randomized studies. Of those, 2 were crossover studies^(^ 37 ^,^ 38 ^)^ and 1 was an open-label study.^(^ 39 ^)^ Comparisons were made between nasal masks and oronasal masks, in 3 studies^(^ 36 ^-^ 38 ^)^; among nasal masks, oronasal masks, and nasal pillows, in 2^(^ 35 ^,^ 39 ^)^; and among nasal masks, oronasal masks, and oral masks, in 1.^(^ 34 ^)^

**Table 1 -:** Studies examining the efficacy of continuous positive airway pressure masks in the treatment obstructive sleep apnea.

Study	Type of study	Patients, n	Types of masks	Baseline AHI, events/h	Residual AHI, events/h	CPAP, mmH_2_O	Performance^a^
Beecroft et al.^(34)^	Observational study	98	N, ON, and O	40.6 ± 25.8	N: 6.7 ± 13.3; ON: 9.8 ± 12.8; O: 10.9 ± 20.1 (ns)	N: 7.7 ± 2.1; ON: 9.7 ± 3.2; O: 8.8 ± 2.0 (ns)	N≈ON≈O
Borel et al.^(35)^	Observational study	2,311	N, ON, and NPs	41.0 ± 21.0	ND	N: ≈ 8.8; ON: ≈ 9.6; NPs:**≈ 8.3 (p < 0.05 for all)	NPs>N>ON
Bettinzoli et al.^(36)^	Observational study	109	N and O	41.1 ± 20.5	N: 2.6 ± 2.5; ON: 4.5 ± 4.4 (p < 0.05)	N: 10.0 ± 2.0; ON: 11.2 ± 2.1 (p < 0.05)	N>ON
Teo et al.^(37)^	Randomized crossover study	24	N and O	47.0 ± 15.2	N: 5.3 ± 3.4; ON: 11.0 ± 10.4 (p = 0.01)	N: 11.4 ± 1.9; ON: 11.8 ± 2.4 (ns)	N>ON
Bakker et al.^(38)^	Randomized crossover study	12	N, ON, and ON+CS	59.8 ± 28.6	N: 0.61 (IR: 1.1); ON: 2.4 (IR: 3.7): ON+CS: 1.7 (IR: 4.0) (p = 0.03 for all)	N: 11.0; ON: 11.1; ON+CS: 11.1 (ns)	N>ON≈ON+CS
Ebben et al.^(39)^	Randomized open-label study	55	N, ON, and NPs	ND	ΔCPAP (ON and N): Moderate OSA: +2.8 ± 2.1; Severe OSA: +6.0 ± 3.2 (p < 0.001)	ND	N≈NPs>ON

AHI: apnea-hypopnea index; CPAP: continuous positive airway pressure; N: nasal mask; ON: oronasal mask; O: oral mask; NPs: nasal pillows; ON+CS: oronasal mask + chin strap; ND: no data; ns: not significant; IR: interquartile range; and OSA: obstructive sleep apnea.

a Performance: summary/conclusion of the study.

The patients included in the 3 observational studies reviewed here had moderate to severe OSA. Beecroft et al.^(^ 34 ^)^ studied 98 patients, who were shown nasal masks, oronasal masks, and an oral mask for CPAP treatment and were allowed to choose one. Most (66%) of the patients chose a nasal mask, whereas 27% chose the oral mask (27%) and 7% chose an oronasal mask. Although the three groups of patients were similar in terms of anthropometric measurements and OSA severity, optimal CPAP (as determined by a CPAP titration study) was on average 2 cmH_2_O higher and the residual AHI was on average 3 events/hour of sleep higher in those who used an oronasal mask than in those who used a nasal mask. Although the difference was not statistically significant, all parameters were worse in those who used an oronasal mask. In addition, one third of the patients who initially chose an oronasal mask chose to change it to a different type of mask during follow-up.^(^ 34 ^)^ The oral mask also showed a trend toward a worse performance, the residual AHI being higher in those who used it than in those who used a nasal mask. Borel et al.^(^ 35 ^)^ conducted an observational cohort study of 2,311 OSA patients who had received a prescription for CPAP treatment and found that nasal masks, oronasal masks, and nasal pillows were used by 62%, 26%, and 11%, respectively. There were statistically significant differences among the three groups of patients regarding CPAP, which was higher in those who used oronasal masks than in those who used nasal masks, being higher in the latter than in those who used nasal pillows. In a multivariate analysis, oronasal masks were associated with subtherapeutic CPAP and low adherence to CPAP treatment (Table 2).^(^ 35 ^)^ Bettinzoli et al.^(^ 36 ^)^ evaluated 109 patients who were allowed to choose between nasal masks (67%) and oronasal masks (42%) for a home titration period of 3-4 nights with an automated CPAP device. Therapeutic CPAP and the residual AHI were significantly higher (+1.2 cmH_2_O and +1.9 events/h, respectively) with the use of an oronasal mask. In a multivariate analysis, the oronasal mask was associated with higher pressure levels.^(^ 36 ^)^ The observational studies reviewed here showed that oronasal masks had the worst performance. The results of those studies should be interpreted with caution because they seem to suggest a potential lack of effectiveness with oronasal mask use. 

In a randomized crossover study, Teo et al.^(^ 37 ^)^ evaluated 24 patients with moderate to severe OSA and no history of oronasal surgery or signs of significant nasal obstruction. The therapeutic CPAP level as determined during titration was similar for nasal and oronasal masks. However, the residual AHI was on average 5.7 events/h higher with the use of an oronasal mask than with the use of a nasal mask (p = 0.01). The standard deviation of the residual AHI was on average 3 times higher with the oronasal mask (10.4 vs. 3.4 events/h), indicating a higher variability in the residual AHI. Arousals and leaks were also greater with the oronasal mask.^(^ 37 ^)^ Bakker et al.^(^ 38 ^)^ evaluated 12 patients with severe OSA and showed that changing from a nasal mask to an oronasal mask significantly increased leak and the residual AHI; however, there was no difference between the two types of masks in terms of the CPAP level. Ebben et al.^(^ 39 ^)^ evaluated 55 patients with mild, moderate, or severe OSA. Patients were randomized to CPAP titration with a nasal mask, an oronasal mask, or nasal pillows. The nasal mask and nasal pillows were similar in terms of CPAP levels. Although the oronasal and nasal masks were similar in terms of the residual AHI, the former required higher pressures than did the latter. This difference increased as the degree of OSA severity increased, being +2.8 ± 2.1 cmH_2_O in patients with moderate OSA and +6.0 ± 3.2 cmH_2_O in those with severe OSA.^(^ 39 ^)^ Therefore, all of the randomized studies reviewed here showed consistent results, showing that the performance of oronasal masks is worse than that of nasal masks. The studies also show that the performance of nasal pillows is similar to that of nasal masks. 

## Impact of the type of mask on adherence to OSA treatment with CPAP

We found 8 studies evaluating the impact of the type of mask on adherence to CPAP treatment (Table 2). As previously mentioned, 2 of the studies describing the impact of the type of mask on adherence to CPAP treatment also included relevant data on the impact of the type of mask on treatment efficacy,^(^ 34 ^,^ 35 ^)^ their characteristics being therefore described in Tables 1 and 2. Of the 8 included studies, 3 were observational studies^(^ 34 ^,^ 35 ^,^ 40 ^)^ and 5 were randomized studies; of those, 4 were crossover studies^(^ 20 ^,^ 41 ^-^ 43 ^)^ and 1 was an open-label study.^(^ 44 ^)^ Comparisons were made between nasal and oronasal masks, in 1 study^(^ 20 ^)^; between nasal masks and nasal pillows, in 2^(^ 42 ^,^ 43 ^)^; between nasal and oral masks, in 2^(^ 41 ^,^ 44 ^)^; among nasal masks, nasal pillows, and oronasal masks, in 2^(^ 35 ^,^ 40 ^)^; and among nasal, oronasal, and oral masks, in 1.^(^ 34 ^)^

**Table 2 -:** Studies examining the impact of the types of masks on adherence to continuous positive airway pressure treatment.

Study	Type of study	Patients, n	Types of masks	Baseline AHI, events/h	Treatment adherence	Performance^a^
Beecroft et al.^(34)^	Observational study	98	N, ON, and O	40.6 ± 25.8	Nights/week in the acclimatization period: N: 5.8 ± 1.7; ON: 3.8 ± 3.0; O: 6.6 ± 0.8 (p < 0.01 for all)	N≈O>ON
Borel et al.^(35)^	Observational study	2,311	N, ON, and NPs	41.0 ± 21.0	N: 5.7 ± 2.2 h/night; ON: 5.1 ± 2.3 h/night (p < 0.0001)	N≈NPs>ON
Bachour et al.^(40)^	Observational study	703	N, ON, and NPs	ND	N: 5.8 ± 2.8 h/night; ON: 4.7 ± 2.8 h/night; NPs: 4.7 ± 3.2 h/night (p < 0.001 for all)	N>NPs≈ON
Mortimore et al.^(20)^	Randomized crossover study	20	N and ON	34.0 ± 5.2	N: 5.3 ± 0.4 h/night; ON: 4.3 ± 0.5 h/night (p = 0.01)	N>ON
Massie et al.^(42)^	Randomized crossover study	39	N and NPs	47.1 ± 35.4	% days of use: N: 85.7 ± 23.5%; NPs: 94.1 ± 8.3% (p = 0.02)	NPs>N
Ryan et al.^(43)^	Randomized crossover study	21	N and NPs	52.4 ± 21.6	N: 5.1 ± 1.9 h/night; NPs: 5.0 ± 1.7 (ns)	NPs≈N
Anderson et al.^(41)^	Randomized crossover study	25	N and O	85.0 ± 36.0	N: 3.8 h/night; O: 3.5 h/night (ns)	N≈O
Khanna et al.^(44)^	Randomized open-label study	38	N and O	N: 63.0 ± 39.0; O: 58.5 ± 34.8	1st month: N: 4.3 ± 2.6 h/night; O: 4.6 ± 2.1 h/night (ns) 2nd month: N: 4.6 ± 2.5 h/night; O: 5.5 ± 2.6 h/night (ns)	N≈O

AHI: apnea-hypopnea index; N: nasal mask; ON: oronasal mask; O: oral mask; NPs: nasal pillows; ND: no data; and ns: not significant.

a Performance: summary/conclusion of the study. Source: Sleep Laboratory, Heart Institute, University of São Paulo School of Medicine Hospital das Clínicas.

The 3 observational studies reviewed here examined a total of 3,112 patients with moderate to severe OSA and showed lower adherence to CPAP treatment with the use of an oronasal mask than with the use of a nasal mask.^(^ 34 ^,^ 35 ^,^ 40 ^)^ Beecroft et al. showed that dropout rates were higher in patients receiving long-term CPAP applied via an oronasal mask than in those receiving long-term CPAP applied via a nasal mask.^(^ 34 ^)^ Treatment adherence was higher with the use of nasal pillows than with the use of nasal masks in one study^(^ 35 ^)^ but lower in another.^(^ 40 ^)^

In a randomized crossover study of 20 patients with moderate to severe OSA, Mortimore et al.^(^ 20 ^)^ initially performed CPAP titration with the use of a nasal mask and subsequently randomized patients to nasal CPAP or oronasal CPAP for 4 weeks each. Adherence to oronasal CPAP was approximately 1 h lower than adherence to nasal CPAP, and 19 of the 20 participants preferred the nasal mask.^(^ 20 ^)^ Oronasal masks have also been associated with poorer sleep quality, less slow-wave sleep, more leaks, less satisfaction, and less comfort when compared with nasal masks.^(^ 37 ^,^ 38 ^,^ 42 ^)^

Two randomized crossover studies compared nasal masks and nasal pillows in terms of adherence to CPAP treatment. Massie et al. evaluated 39 OSA patients using nasal masks and nasal pillows for 3 weeks each and found that treatment adherence was significantly higher with the use of nasal pillows.^(^ 42 ^)^ Ryan et al. studied 21 severe OSA patients using nasal masks and nasal pillows for 4 weeks each.^(^ 43 ^)^ The authors found no differences between the two types of CPAP masks in terms of their impact on treatment adherence. However, the participants complained of nasal congestion, nasal dryness, nosebleeds, and headaches more frequently when they used nasal pillows than they did when they used nasal masks.^(^ 43 ^)^ Two studies showed that oral and nasal masks were similar in terms of their impact on treatment adherence.^(^ 41 ^,^ 44 ^)^ However, oral masks are not widely accepted and are rarely used in clinical practice. 

## Final considerations

We conclude that the type of mask can influence the efficacy of and adherence to CPAP treatment in patients with OSA. Nasal pillows constitute an alternative to nasal masks and appear to be effective in the treatment of OSA. Nasal pillows are lighter, and their initial acceptance might be higher. However, they can cause more nasal problems, particularly when a CPAP > 12 cmH_2_O is used. A recent study showed that nasal pillows can be used even at pressures ≥ 12 cmH_2_O.^(^ 45 ^)^ Oral masks appear to be effective in the treatment of OSA because they hold the tongue in place with a tongue guide; however, they are rarely used in clinical practice because their level of acceptance is low. Several theoretical and experimental studies have shown that oronasal masks can affect the efficacy of and adherence to OSA treatment with CPAP.^(^ 20 ^,^ 26 ^,^ 31 ^,^ 36 ^,^ 37 ^)^ In comparison with nasal masks, oronasal masks often require higher CPAP levels and are associated with a higher residual AHI and lower adherence to treatment. How can we treat OSA patients who breathe through their mouth either by habit or because of nasal obstruction? We believe that the first step is to treat their nasal obstruction, either clinically or surgically. Another important point is that mouth breathing does not necessarily mean that nasal masks are contraindicated. For example, there is evidence that the use of nasal CPAP leads to a change of habit, reducing mouth opening and the number of oral breaths.^(^ 40 ^,^ 46 ^,^ 47 ^)^ However, many patients adapt well to oronasal masks and show perfect OSA control. Our review suggests two conclusions: first, nasal interfaces (i.e., nasal masks and nasal pillows) should always be the first choice; second, patients using oronasal masks must be monitored because the risks of CPAP treatment failure, nonadherence, and discontinuation are higher. Further studies are needed in order to understand the exact mechanisms by which oronasal interfaces affect the efficacy of OSA treatment with CPAP. 
